# Supplementary material for: Resting State fMRI Functional Connectivity-Based Classification Using a Convolutional Neural Network Architecture
Source: Front Neuroinform. 2017 Oct 17;11:61. doi: 10.3389/fninf.2017.00061 (PMC5651030; doi:10.3389/fninf.2017.00061)
Supplement: Supplementary file 1 [file Image_1.pdf]

Supplementary Material

Resting state fMRI functional connectivity-based classification using a convolutional neural network architecture

Regina Meszlényi, Krisztian Buza and Zoltán Vidnyánszky

Correspondence: Regina Meszlényi: meszlenyi.regina@ttk.mta.hu

1 Dynamic Time Warping distance and warping path length

Dynamic Time Warping is a time-series distance measure that can correct for even dynamically changing phase-differences between signals. DTW was first applied in the field of speech recognition (Sakoe and Chiba, 1978). DTW distance can be efficiently used for time-series classification (Ding et al., 2008; Xi et al., 2006), and recently our group has demonstrated its potential for fMRI data analysis (Meszlényi et al., 2016a, 2016b, 2017).

DTW is a so called edit distance, which means that it measures the "cost" of transforming one time-series to the other one (Suppl. Fig. 1. A). Two editing steps are possible for transforming two time-series x_1 and x_2: replacement of an element of x_1 to an element of x_2 or an elongation of an element in x_1 or x_2. The cost of each editing step is the difference of the matched elements, while the overall cost of transformation is the sum of the costs of each editing step: DTW distance is the minimal possible transformation cost. We can calculate DTW distance of x_1 and x_2 with length of l_1 and l_2 by filling the entries of an l_1 x l_2 matrix column-by-column or row-by-row (Suppl. Fig. 1. B,C) based on Equation 1:

Eq 1.: $$\mathrm{DTW(i,j)} = \begin{cases} \|\mathrm{x_1(i), x_2(j)}\| + \min\{\mathrm{DTW(i, j-1), DTW(i-1, j), DTW(i-1, j-1)}\} \text{ if } \mathrm{i, j} > 1 \\ \|\mathrm{x_1(i), x_2(j)}\| + \mathrm{DTW(i, j-1)} \text{ if } \mathrm{i} = 1, \mathrm{j} > 1 \\ \|\mathrm{x_1(i), x_2(j)}\| + \mathrm{DTW(i-1, j)} \text{ if } \mathrm{j} = 1, \mathrm{i} > 1 \\ \|\mathrm{x_1(i), x_2(j)}\| \text{ if } \mathrm{i} = 1, \mathrm{j} = 1 \end{cases}$$

where $x_1(i)$ denotes the i-th value in time series x_1 and $x_2(j)$ denotes the j-th value in time series x_2 and $DTW(l_1,l_2)$ is the DTW-distance of the two time series (Suppl. Fig. 1. D). To constrain the maximal allowed phase-difference (time-shift between matched elements) and to speed-up DTW-calculations, we can calculate only those entries of the DTW matrix that are close to the main diagonal (Sakoe and Chiba, 1978). The maximal allowed time-shift is the so called warping window (Suppl. Fig. 1. B).

After filling-in the DTW matrix, one can reconstruct which editing steps led to the minimal DTW distance value, i.e. we can determine the optimal matching between each element of the two time series (Suppl. Fig. 1. E): this optimal matching sequence is called the warping path (Suppl. Fig. 1. B). When comparing identical time-series the warping path exactly follows the main diagonal (no elongation steps are necessary), while phase-differences between signals will introduce elongation steps, therefore the length of the warping path will increase compared to the main diagonal. The difference between the warping path length and the length of the main diagonal can characterize the overall phase difference and the stability of this time-delay structure between the two time-series (Meszlényi et al., 2016a) and this measure is referred as warping path length throughout the paper.

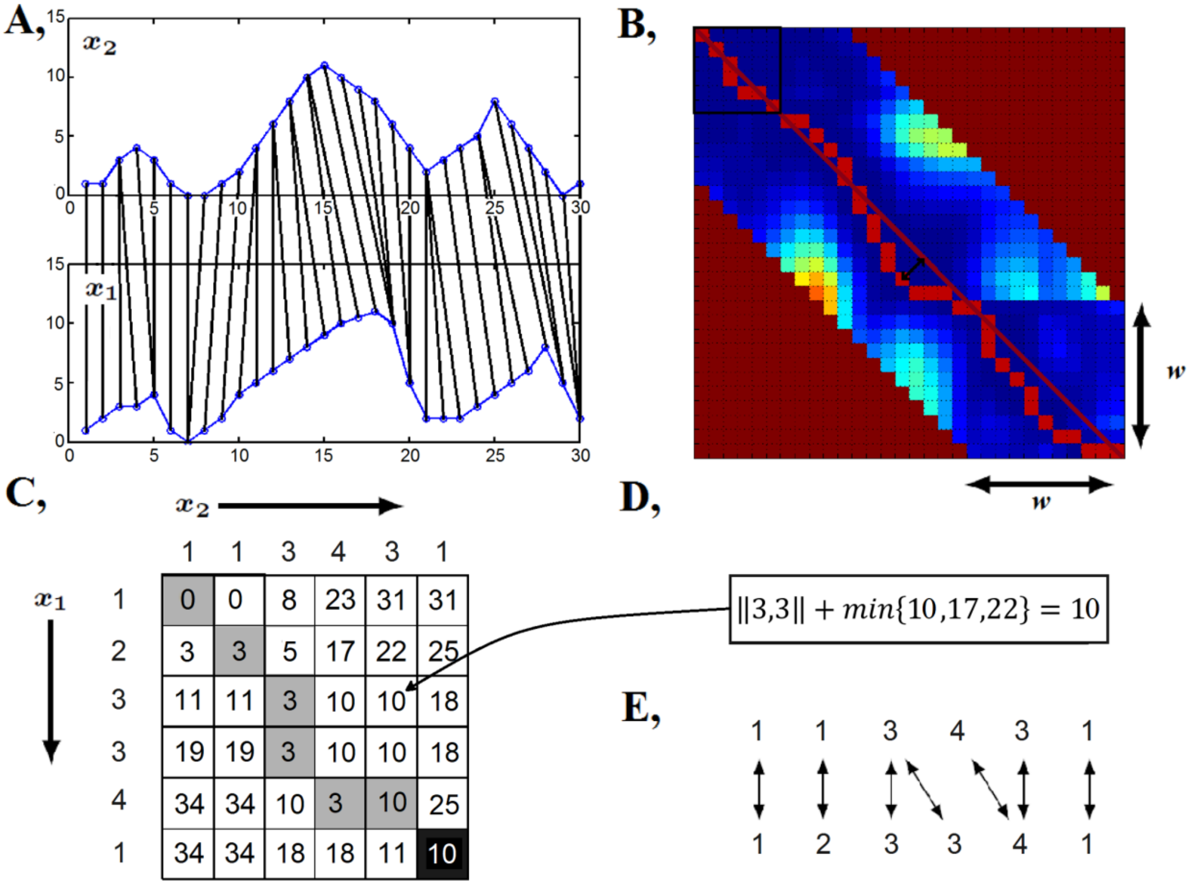

Supplementary Figure 1.: Figure adapted from (Meszlényi et al., 2017) A, x_1 and x_2 time series compared with DTW: the i-th element of x_1 is elastically matched to the appropriate element of x_2. B, The filled-out DTW matrix plotted as a heat-map (hot colors represent larger values), w denotes the size of the warping window, the maximal allowed time-lag between two matched time series element. The main diagonal is represented by the dark red line, while the optimal warping path is plotted with red. The time-delay between the x_1 and x_2 time series at a given time-point is given by the warping path's deviation from the main diagonal (represented by the black arrow) C, Calculation of DTW distance by filling out the DTW matrix: the example shows the first six element of x_1 and x_2 time series (highlighted with the black rectangle in Fig… .B). Elements of x_1 corresponds to rows, while elements of x_2 corresponds to columns of the matrix. The optimal warping path is highlighted with dark grey. D, Formula to calculate entry (i,j) – in this example entry (3,5): squared distance of $x_1(i)$ and $x_2(j)$ plus the minimum of the matrix entries (i-1,j), (i-1,j-1), (i,j-1) E, Optimal matching of the first six elements of x_1 and x_2 revealed by the DTW matrix.

2 Results of the traditional machine learning methods

We also tested how traditional machine learning methods perform compared to the CCNN method. We conducted experiments with two algorithms that can handle the curse of dimensionality with feature selection, namely a linear SVM classifier combined with ANOVA F-test based feature selection described in (Abraham et al., 2014) and a LASSO model also frequently used in fMRI based classification (Li* et al., 2009; Rosa et al., 2015; Ryali et al., 2010). Our adopted approach is detailed in (Meszlényi et al., 2016b).

2.1 Results on the combined DTW distance and warping path length dataset

As the CCNN method's best result was achieved on the combination of two connectivity features, DTW distance and path length, we calculated baseline accuracies on this dataset with different hyper-parameter setups of both SVM and LASSO models. Based on the results presented in Supplementary Table 1 and 2 we can conclude that neither of these methods could achieve better performance than the CCNN model. We also note that the resulting predictions from different hyper-parameter setups do not differ significantly ($p>0.15$ in all cases).

2.1.1 SVM results

As all our neural network models (including the CCNN architecture) are trained to extract 128 features from the connectivity data, we selected the best 128 connectivity features based on the ANOVA F-tests, and performed linear SVM classification on the selected features with five different values of the complexity parameter C. The accuracy values are summarized in Supplementary Table 1:

C	1	0.5	0.1	0.05	0.01
Accuracy (%)	62.3	61.6	66.4	66.4	63.0

Supplementary Table 1: Results of the linear SVM classification

2.1.2 LASSO results

In case of the LASSO model based classification, we ran experiments with six values of the regularization hyper-parameter λ to enforce the selection of approximately 128 connectivity features. Supplementary Table 2 summarizes the accuracy values of the resulting classifications as well as the mean and standard deviation of the number of selected features in the seven folds of cross-validation.

λ	0.001	0.0009	0.0008	0.0007	0.0006	0.0005
No. features (mean±std)	119±2.1	121±2.6	122±3.5	123±3.5	125±3.4	127±4.4
Accuracy (%)	69.2	69.9	69.9	69.2	68.5	69.2

Supplementary Table 2: Results of the LASSO classification

2.2 Results on the single datasets

The CCNN method achieved considerably better accuracy on the combined dataset than on any dataset containing only a single connectivity feature, therefore it is also important to examine whether SVM and LASSO models can similarly well utilize information from multiple connectivity features. Consequently we conducted classification experiments with SVM and LASSO models based on the three single connectivity feature datasets: correlation, DTW distance and warping path length.

2.2.1 SVM Results

Similarly to the training combined dataset, we selected the best 128 connectivity features based on the ANOVA F-tests, and performed linear SVM classification on the selected features with C = 0.05. The accuracy values are summarized in Supplementary Table 3:

C=0.05	CORR	DTW	Path length	*DTW+Path length*
Accuracy (%)	54.1	67.1	64.4	*66.4*

Supplementary Table 3: Results of the linear SVM classification

2.2.2 LASSO results

We performed LASSO classification on the three single dataset with $\lambda = 0.0008$. The results are summarized in Supplementary Table 4:

λ =0.0008	CORR	DTW	Path length	*DTW+Path length*
Accuracy (%)	60.3	59.6	69.9	*69.9*

Supplementary Table 4: Results of the LASSO classification

2.3 Discussion of the results of the traditional algorithms

On the combined DTW distance and warping path length dataset the best SVM accuracy is 5.5% lower than the CCNN result, while in case of the LASSO classifiers, the best performing model has 2% lower accuracy than the CCNN model, however neither of these differences are significant ($p>0.1$). Since we ran experiments with different hyper-parameters, our estimates of these test-performances might be over-optimistic. Even with this slight positive bias none of these classifiers achieved better results than the CCNN model.

Most importantly we note that neither the SVM, nor the LASSO model was able to utilize additional information from the combination of different connectivity features, i.e. the SVM classifier even achieved slightly better (0.6% higher) accuracy on the single DTW distance dataset than on the combined data and the LASSO model has the same result on the single path length and the combined datasets.

Our results confirm that while the convolutional model is able to integrate information from multiple different connectivity features, traditional machine learning algorithms do not gain performance from the additional information. Furthermore, based on current deep learning research one can assume that both with growing number of measurements and with growing number of measured dimensions, i.e. in case of increasingly complex data, the difference between the performance of traditional machine learning models and deep learning methods increase (Goodfellow et al., 2016; LeCun et al., 2015). Therefore the proposed CCNN architecture can hold great potential for future research.

3 References

Abraham, A., Pedregosa, F., Eickenberg, M., Gervais, P., Mueller, A., Kossaifi, J., et al. (2014). Machine learning for neuroimaging with scikit-learn. *Front. Neuroinformatics* 8. doi:10.3389/fninf.2014.00014.

Ding, H., Trajcevski, G., Scheuermann, P., Wang, X., and Keogh, E. (2008). Querying and Mining of Time Series Data: Experimental Comparison of Representations and Distance Measures. *Proc VLDB Endow* 1, 1542–1552. doi:10.14778/1454159.1454226.

Goodfellow, I., Bengio, Y., and Courville, A. (2016). *Deep Learning*. Cambridge, Massachusetts: The MIT Press.

LeCun, Y., Bengio, Y., and Hinton, G. (2015). Deep learning. *Nature* 521, 436–444. doi:10.1038/nature14539.

Li*, Y., Namburi, P., Yu, Z., Guan, C., Feng, J., and Gu, Z. (2009). Voxel Selection in fMRI Data Analysis Based on Sparse Representation. *IEEE Trans. Biomed. Eng.* 56, 2439–2451. doi:10.1109/TBME.2009.2025866.

Meszlényi, R. J., Hermann, P., Buza, K., Gál, V., and Vidnyánszky, Z. (2017). Resting State fMRI Functional Connectivity Analysis Using Dynamic Time Warping. *Front. Neurosci.* 11. doi:10.3389/fnins.2017.00075.

Meszlényi, R., Peska, L., Gál, V., Vidnyánszky, Z., and Buza, K. (2016a). A Model for Classification Based on the Functional Connectivity Pattern Dynamics of the Brain. in *2016 Third European Network Intelligence Conference (ENIC)* (Wrocław), 203–208. doi:10.1109/ENIC.2016.037.

Meszlényi, R., Peska, L., Gál, V., Vidnyánszky, Z., and Buza, K. (2016b). Classification of fMRI data using dynamic time warping based functional connectivity analysis. in *2016 24th European Signal Processing Conference (EUSIPCO)* (Budapest), 245–249. doi:10.1109/EUSIPCO.2016.7760247.

Rosa, M. J., Portugal, L., Hahn, T., Fallgatter, A. J., Garrido, M. I., Shawe-Taylor, J., et al. (2015). Sparse network-based models for patient classification using fMRI. *NeuroImage* 105, 493–506. doi:10.1016/j.neuroimage.2014.11.021.

Ryali, S., Supekar, K., Abrams, D. A., and Menon, V. (2010). Sparse logistic regression for whole-brain classification of fMRI data. *NeuroImage* 51, 752–764. doi:10.1016/j.neuroimage.2010.02.040.

Sakoe, H., and Chiba, S. (1978). Dynamic programming algorithm optimization for spoken word recognition. *IEEE Trans. Acoust. Speech Signal Process.* 26, 43–49. doi:10.1109/TASSP.1978.1163055.

Xi, X., Keogh, E., Shelton, C., Wei, L., and Ratanamahatana, C. A. (2006). Fast Time Series Classification Using Numerosity Reduction. in *Proceedings of the 23rd International Conference on Machine Learning* ICML ’06. (New York, NY, USA: ACM), 1033–1040. doi:10.1145/1143844.1143974.
