# Supplementary material for: Resting State fMRI Functional Connectivity-Based Classification Using a Convolutional Neural Network Architecture
Source: Front Neuroinform. 2017 Oct 17;11:61. doi: 10.3389/fninf.2017.00061 (PMC5651030; doi:10.3389/fninf.2017.00061)
Supplement: Supplementary file 2 [file Image_2.PDF]

Supplementary Material

Resting state fMRI functional connectivity-based classification using a convolutional neural network architecture

Regina Meszlényi, Krisztian Buza and Zoltán Vidnyánszky

Correspondence: Regina Meszlényi: meszlenyi.regina@ttk.mta.hu

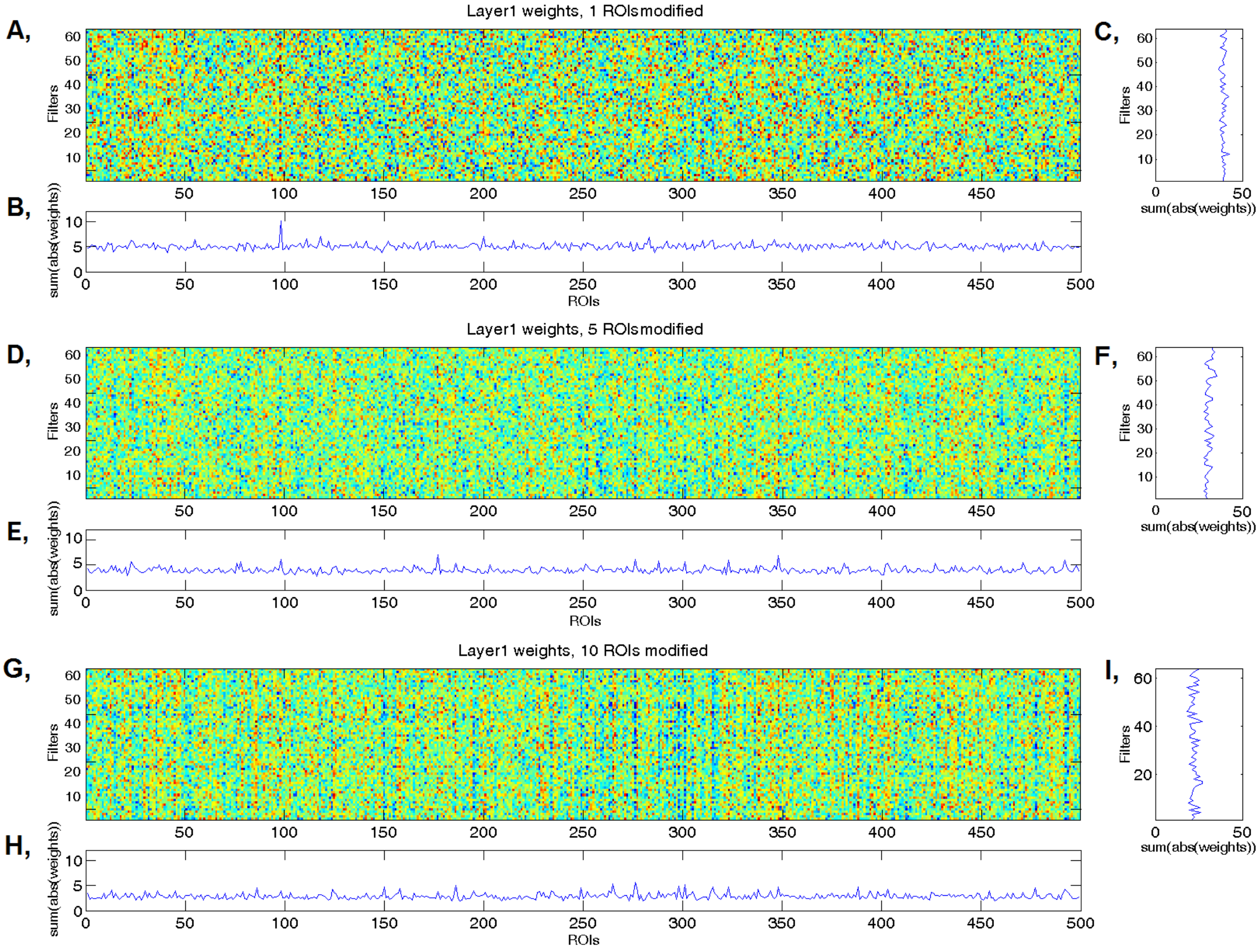

Supplementary Figure 2: A-C, Learned weights of the first convolutional layer of the CCNN model trained on the whole simulated dataset, with one ROI modification. A, Colormap of the 64x499 weights of the first convolutional layer. B, To determine which ROIs play important role in the classification, we summarized the absolute values of the weights through the 64 filters. High values represent ROIs that have significant effect in most filters: the sole highest peak is indeed identical to the one altered ROI C, To determine which filters are the most effective, we summarized the absolute values of weights through the 499 ROIs. High values represent filters that have substantial influence on the output. D-E, Learned weights of the first convolutional layer of the CCNN model trained on the whole simulated dataset, with five ROI modification. D, Colormap of the 64x499 weights of the first

convolutional layer. E, Summarized absolute values of the weights through the 64 filters. Four of the five highest peaks overlap with the modified five ROIs. F, Summarized absolute values of weights through the 499 ROIs. G-I, Learned weights of the first convolutional layer of the CCNN model trained on the whole simulated dataset, with ten ROI modification. G, Colormap of the 64x499 weights of the first convolutional layer. H, Summarized absolute values of the weights through the 64 filters. Four of the ten highest peaks overlap with the modified ROIs. I, Summarized absolute values of weights through the 499 ROIs.

frontiers
